# Sarcopenia as a Risk Factor in Patients Undergoing Transjugular Intrahepatic Portosystemic Shunt (TIPS) Implantation

**DOI:** 10.3390/diagnostics15111440

**Published:** 2025-06-05

**Authors:** Laura Büttner, Annette Aigner, Regina Stegherr, Simon Iseke, Martin Jonczyk, Willie Magnus Lüdemann, Timo Alexander Auer, Federico Collettini, Dirk Schnapauff, Maximilian de Bucourt, Bernhard Gebauer, Dominik Geisel, Georg Böning

**Affiliations:** 1 Department of Radiology, Charité—Universitätsmedizin Berlin, Corporate Member of Freie Universität Berlin and Humboldt Universität zu Berlin, Charitéplatz 1, 10117 Berlin, Germany; 2 Institute of Biometry and Clinical Epidemiology, Charité—Universitätsmedizin Berlin, Corporate Member of Freie Universität Berlin and Humboldt Universität zu Berlin, Charitéplatz 1, 10117 Berlin, Germany; 3 Department of Diagnostic and Interventional Radiology, Pediatric Radiology and Neuroradiology, Rostock University Medical Center, 18057 Rostock, Germany; 4 Berlin Institute of Health at Charité—Universitätsmedizin Berlin, Charitéplatz 1, 10117 Berlin, Germany

**Keywords:** body composition, computed tomography, transjugular intrahepatic portosystemic shunt, sarcopenia

## Abstract

**Background**: Prior studies suggest that patients’ body composition changes following transjugular intrahepatic portosystemic shunt (TIPS) implantation, potentially influencing complications and survival. **Method**: A prototype artificial intelligence (AI)-based, automated computed tomography (CT) body composition analysis tool was used to assess body composition imaging parameters in pre- and postinterventional scans of TIPS patients: visceral (VAT) and subcutaneous adipose tissue (SAT) areas, psoas muscle area (PMA), and total abdominal muscle area (TAMA). Sarcopenia was defined as a lumbar skeletal muscle index (LSMI) ≤ 38.5 cm^2^/m^2^ in women and ≤52.4 cm^2^/m^2^ in men. We analyzed longitudinal changes in body composition and investigated the impact of sarcopenia at TIPS implantation on the risk of TIPS thrombosis, hepatic encephalopathy, complications, and death using Cox regression models. **Results**: No clear trend emerged regarding changes in body composition parameters during postinterventional follow-up. Sarcopenia at TIPS implantation increased the instantaneous risk of postinterventional complications (hazard ratio (HR) 1.67; 95% confidence interval (CI) 0.95–2.93), development of hepatic encephalopathy (HR 1.65; 0.81–3.33), as well as the risk of dying within one year (HR 1.39; 0.66–2.92). **Conclusions**: CT body composition analysis may help in identifying high-risk patients undergoing TIPS implantation. Sarcopenia was associated with increased mortality and a higher incidence of postinterventional complications, particularly hepatic encephalopathy.

## 1. Introduction

Transjugular intrahepatic portosystemic shunt (TIPS) insertion has been increasingly used to treat the symptoms of portal hypertension, primarily refractory ascites, and variceal bleeding, and is now considered the gold standard among interventional/surgical options [1,2]. Moreover, TIPS can help improve catabolism, thereby contributing to muscle regeneration and the reduction in visceral fat [3]. Studies indicate that an increase in muscle mass improves both long-term outcome and survival after TIPS implantation, since sarcopenic individuals have a higher risk of mortality [4]. Sarcopenia is characterized by a loss of skeletal muscle mass, strength, and function. Several factors are involved in the development of sarcopenia: dietary intake is often insufficient to meet energy expenditure, absorption is impaired, and substrate utilization is disturbed due to liver disease [5,6]. Sarcopenia has also been associated with a higher rate of cirrhosis-related complications, such as hepatic encephalopathy (HE), ascites, or infections, and impairs patients’ quality of life [7,8]. Artificial intelligence (AI)-based computed tomography (CT) body composition has already been used in several studies to investigate the impact of sarcopenia on patients’ outcomes, for example, in cardiovascular or oncological diseases [9,10,11,12,13]. CT body composition analysis uses CT scans to quantify skeletal muscle mass since muscle atrophy is characterized by both a smaller muscle size and a higher proportion of intermuscular and intramuscular fat (myosteatosis) [14]. Compared to manual segmentation of CT data, automated tissue segmentation allows analysis of larger data sets [9] and is recommended for evaluating sarcopenia in the most recent version of the European Association for the Study of the Liver clinical practice guidelines [15]. The skeletal muscle area (SMA) and skeletal muscle index (SMI) can be derived from CT scans and have become the standard for evaluating sarcopenia [15,16]. Since CT scans are acquired in the follow-up of many therapeutic regimes, they can also be used to monitor dynamic changes in skeletal muscle and fat mass in response to interventions and treatments.

Therefore, the aim of this study was to evaluate the imaging parameters of body composition derived from automatically segmented CT scans as potential risk factors for complications, TIPS thrombosis, development of hepatic encephalopathy, and death following TIPS implantation.

## 2. Materials and Methods

Study Population: Retrospectively, 835 patients were included who underwent TIPS treatment at our center between June 1993 and December 2018. All implantations were analyzed with at least one CT examination before TIPS implantation (baseline, maximum of 6 months prior implantation) or CT examinations after TIPS implantation (minimum of 6 months, maximum of two follow-up scans). CT scans were excluded if they were unsuitable, e.g., if lumbar vertebral body levels were not displayed. Preinterventional imaging was usually performed to rule out hepatocellular carcinoma in patients with cirrhosis, because of abdominal pain or for intervention planning.

Additionally, demographic information, clinical data, laboratory results, and procedural parameters were collected. Patients were observed until the end of data collection in 2018, or, if occurred before, death, liver transplantation, last recanalization attempt, iatrogenic TIPS occlusion, or last patient contact during the observation period. In cases where a patient experienced two consecutive complications, e.g., postinterventional bleeding with an infection occurring later (e.g., pneumonia), the time interval until the first complication was considered. The same approach was used for patients who suffered a recurrence of dysfunction after TIPS thrombosis with appropriate therapy. We defined hepatic encephalopathy (HE) as either a newly developed episode of overt HE or a documented clinical worsening of pre-existing HE.

This retrospective study was approved by the hospital’s ethics committee (EA4/085/17).

Technique of TIPS: TIPS was performed as previously described [17], with treatment decisions made through an interdisciplinary case review involving hepatologists, radiologists, and surgeons.

**Figure 1:**
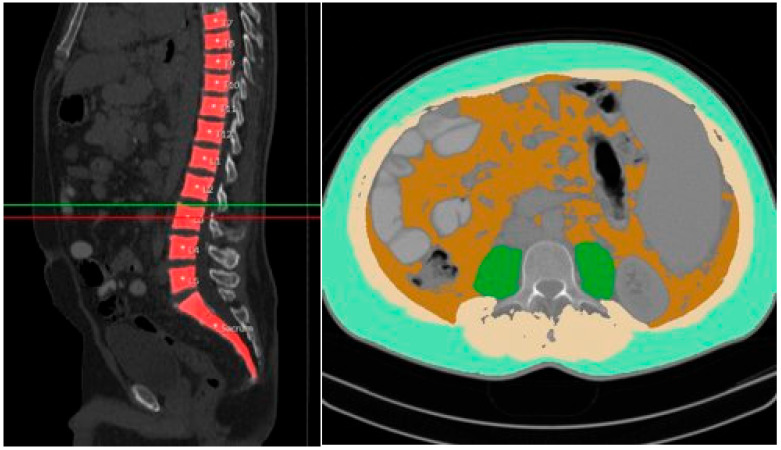
Example of semiautomated segmentations of subcutaneous adipose tissue (SAT; pale green), visceral adipose tissue (VAT; brown), psoas muscle area (PMA; green), and total abdominal muscle area (TAMA, beige + green) at L3 level (indicated by green and red line).

CT Body Composition Analysis: A picture archiving and communications system (PACS)-integrated, AI-based software tool (Visage version 7.1, Visage Imaging GmbH, Berlin, Germany), using a convolutional neural network, was used as previously described [10]. For analysis, 5 mm thick Digital Imaging and Communications in Medicine (DICOM) slice files at the mid-L3 level were processed. Semiautomatic segmentation of the following tissues was performed on single-slice images to quantify psoas muscle area (PMA), total abdominal muscle area (TAMA), visceral adipose tissue (VAT), and subcutaneous adipose tissue (SAT) (Figure 1). All automatic segmentations were reviewed by an experienced radiologist. Manual corrections were performed in a small number of cases to prevent misclassification, for example, to exclude hypodense stool in the intestines that could be mistakenly identified as body fat.

The abdominal adipose tissue ratio (ATR) was calculated as the ratio of VAT to SAT. The lumbar skeletal muscle index (LSMI) at L3 was derived by normalizing the TAMA to the patient’s body height: TAMA/body height^2^. Sarcopenia was defined as LSMI ≤ 38.5 cm^2^/m^2^ in women and ≤52.4 cm^2^/m^2^ in men [9,18], while sarcopenic obesity as sarcopenia in combination with a Body Mass Index (BMI) ≥ 30 [9,19].

Statistical analysis: We report medians along with interquartile ranges (IQR) for baseline body composition parameters, absolute and relative frequencies of all outcome parameters, stratified by sarcopenia and sarcopenic obesity at baseline. The most relevant body composition parameters are displayed longitudinally with reference to the time of TIPS implantation by scatter and spaghetti plots, along with a locally weighted scatterplot smoothing (LOESS) estimate.

Time to death is plotted using Kaplan–Meier curves, along with 95% confidence intervals (CIs). Incidence rates per 100 person-years are reported for all outcome parameters along with 95% CIs, stratified by sarcopenia at baseline. To assess the association between sarcopenia or the LSMI at baseline with time to death, we used Cox proportional hazards regression models. For the other outcomes of hepatic encephalopathy (HE), thrombosis, and complication, Cox regression models accounting for the competing event death were used. Cause-specific hazard ratio (HR) estimates for the event and the competing event were derived. All models were run with and without adjustment for potential confounders, and HR estimates are displayed with 95% CIs. Confounders (age, sex, ascites before TIPS, and performance status) were selected based on clinical experience and literature research. Statistical analyses and data handling were performed with R and additional R packages [20,21,22,23].

## 3. Results

Basic demographic data of the study population have been previously published [17]. According to our definition, 135 patients were classified as sarcopenic, of which 22 patients were classified as sarcopenic obese. Median lumbar skeletal muscle index (LSMI) was 41.8 cm^2^/m^2^ and the adipose tissue ratio (ATR) was 0.7 (Table 1).

Peri- and postinterventional complications such as infections or bleeding were more common in patients with sarcopenia (44.4% vs. 28.3%), but not in patients with sarcopenic obesity. In patients with complications, the median LSMI was lower (40.6 cm^2^/m^2^ vs. 42.6 cm^2^/m^2^). HE was more common in patients with sarcopenia (26.7% vs. 16.7%), and the LSMI was very similar for patients with HE compared to those who did not develop HE (41.7 cm^2^/m^2^ vs. 42.0 cm^2^/m^2^). TIPS dysfunction due to stenosis or thrombosis of the stent was less common in patients with sarcopenia (20.7% vs. 26.7%). Yet, TIPS disfunction was more common in patients with sarcopenic obesity than in patients without (40.9% vs. 19.9%) (see also Table 2).

**Figure 2:**
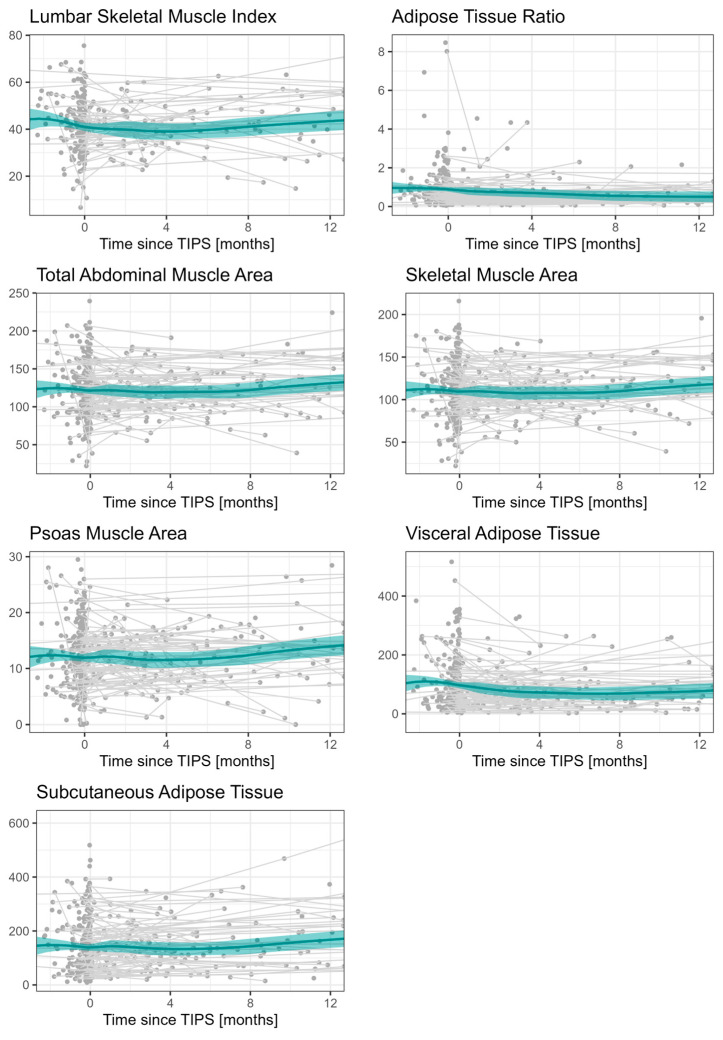
Development of body composition imaging parameters over time; plots are limited to 2 months before TIPS and 12 months after TIPS, but all data are included in the LOESS estimator. Body composition parameters (LSMI, ATR, TAMA, SMA, PMA, VAT, SAT) show no clear trend after TIPS implantation.

Body composition parameters (LSMI, ATR, TAMA, SMA, PMA, VAT, SAT) themselves show no clear trend after TIPS implantation. The data are presented for the period 2 months before and 12 months after TIPS implantation in order to ensure a clearer visualization, as the observed time points after TIPS implantation were very heterogeneous (Figure 2).

**Figure 3:**
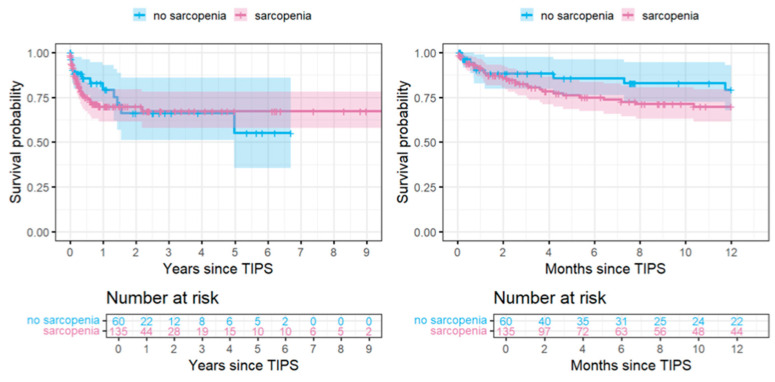
Survival probability shown as Kaplan–Meier curves along with 95% confidence intervals (CIs) for the whole observation period, just as for the first year after TIPS implantation, stratified by sarcopenia at baseline. The Kaplan–Meier curves show a higher mortality in sarcopenic patients within the first 1 year or 1.5 years.

**Figure 4:**
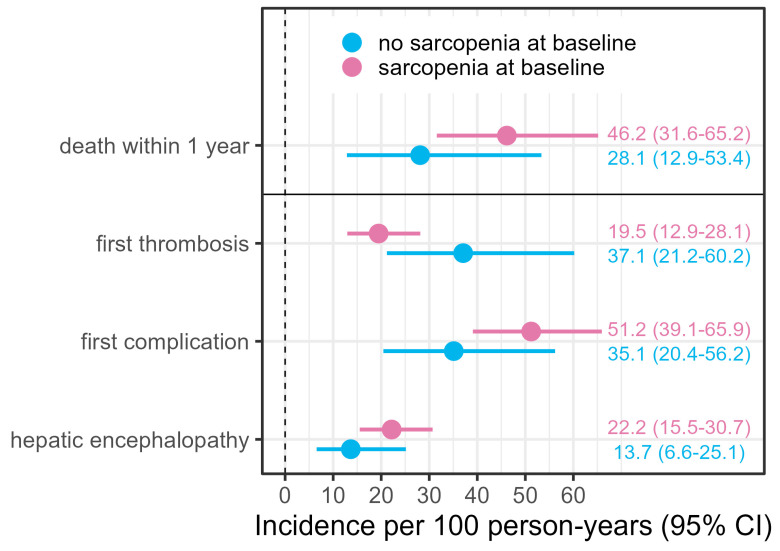
Incidence rates per 100 person-years for all outcome parameters along with 95% confidence intervals, stratified by sarcopenia at baseline. The incidence of death, complication, and HE, but not of thrombosis is increased in patients who are sarcopenic at baseline (i.e., at TIPS).

The Kaplan–Meier curves show a higher mortality in sarcopenic patients within the first 1 year or 1.5 years. Within this first year after TIPS, the incidence of mortality in sarcopenic patients was 46 per 100 person-years compared to about 28 in non-sarcopenic patients. Within the later observational period, this trend changes, such that the Cox proportional hazards assumption might not be fulfilled for the whole time period. Furthermore, the incidences of HE and complication are higher for sarcopenic patients throughout the whole observation period, whereas the incidence of thrombosis is reduced (Figure 3 and Figure 4).

**Figure 5:**
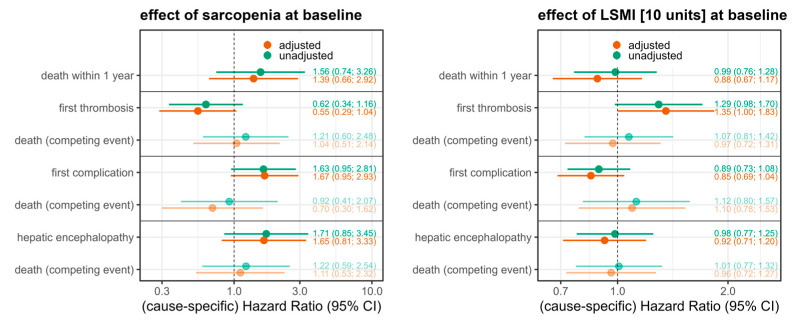
Unadjusted and adjusted hazard ratio estimates derived from Cox regression (for death within 1 year) and competing risk Cox regression models (all other), along with 95% confidence intervals (CIs). For the outcome variables HE, thrombosis, and complication, cause-specific hazard ratio estimates are displayed along with the effect estimates for the competing event of death. Sarcopenia at baseline (i.e., at TIPS) increases the hazards of death, complication, and HE but reduces the hazards of thrombosis. Adjusted and unadjusted effects are rather similar. The results for the LSMI are in line with these findings.

We estimated that sarcopenic patients have about 1.6-fold increased hazards of death within the first year (HR = 1.56; 95% CI 0.74–3.26), where this effect is only slightly reduced given adjustment for confounders (HR = 1.39; 0.66–2.92). Sarcopenic patients also have about 1.6-fold increased cause-specific hazards of a first complication after TIPS (cause-specific HR = 1.67; 0.95–2.81), also independent of confounders. The effect estimate for the competing event death (cause-specific HR 0.70; CI 0.30–1.62) indicates that the hazards for death without prior complication are reduced due to sarcopenia, which means that patients often have a complication before they die. In line with this finding, an LSMI increase of 10 units lowered the hazards of postinterventional complications (cause-specific HR 0.85, CI 0.69–1.04). The hazards of a first thrombosis after TIPS are reduced if the patient was sarcopenic at baseline, about 0.6-fold (cause-specific HR = 0.62; CI 0.34–1.16), where this effect slightly increases when adjusted for the confounders. The effect estimate for the competing event death indicates that there is no influence regarding the risk of death if there was no thrombosis before (cause-specific HR 1.04 CI 0.51–2.14). Sarcopenia was also associated with an increased instantaneous risk of postinterventional HE (cause-specific HR 1.65; CI 0.81–3.33). An increase in the LSMI by 10 units lowered a patient’s risk of developing postinterventional HE (cause-specific HR 0.92; CI 0.71–1.20) (Figure 5). Due to the low number of observations and events, the estimation of the association between sarcopenia or the LSMI and the outcome parameters is very imprecise, reflected in wide confidence intervals.

## 4. Discussion

The present study investigated the use of AI-based CT body composition analysis in pre- and postinterventional CT scans to assess body composition imaging parameters, including the visceral adipose tissue (VAT) and subcutaneous adipose tissue (SAT) area, psoas muscle area (PMA), and total abdominal muscle area (TAMA). Additionally, we analyzed the association between these imaging parameters and survival, as well as the occurrence of postinterventional complications, TIPS thrombosis, and hepatic encephalopathy in patients undergoing TIPS procedures.

In our analyses, we observed no clear trend regarding changes in body composition parameters (PMA, TAMA, VTA, and SAT) within one year after TIPS implantation. Lui et al. reported an increase in the skeletal muscle area, skeletal mass index, and fat mass indicators five months post-TIPS, which were preserved for one year [5]. Other studies have also documented increases in body weight and muscle mass following TIPS [24,25]. These changes may result from improved dietary intake, enhanced nutrition absorption, and better metabolic function after TIPS [14,26]. By alleviating the symptoms of portal hypertension, especially by reducing ascites, patients have more appetite and improved nutrient absorption once their ascites and other symptoms of portal hypertension subside [27].

We found sarcopenia at TIPS implantation to be associated with a higher postinterventional mortality (HR = 1.56). Similarly, previous studies have identified sarcopenia as a predictor of poorer prognosis in patients with cirrhosis [5,28,29,30], likely due to poor nutritional and physical status independent of liver function [31]. Further supporting the impact of a patient’s nutritional status on survival, TIPS implantation has been reported to reverse sarcopenia and improve survival [5]. Given the high prevalence of sarcopenia in liver cirrhosis, particularly in decompensated cases (up to 50%), body composition parameters may help identify patients requiring closer monitoring [15,16]. However, some studies do not report increased mortality in sarcopenic patients [32], suggesting that a diagnosis of sarcopenia alone should not be considered a contraindication for TIPS.

In our study, sarcopenia was associated with an increased instantaneous risk of postinterventional complications, such as infection or bleeding (HR = 1.63). Similarly, previous research reported sarcopenia to be associated with severe complications, including acute-on-chronic liver failure in decompensated liver cirrhosis [33]. Hepatic encephalopathy (HE), one of the most concerning complications of the procedure, results from the shunting of portal blood into the systemic circulation after TIPS [6]. The overall incidence of HE after TIPS ranges from 25% to 45%, while a lower range of 13–36% is reported when only new or exacerbated HE is considered [34]. Two key factors contribute to HE development post-TIPS: shunt volume and pre-TIPS hepatic function. Our findings confirm sarcopenia as a risk factor for the development of post-TIPS HE (HR = 1.71), consistent with the results of previous studies [35,36]. Skeletal muscle plays a compensatory role in ammonia metabolism and degradation, through the enzyme glutamine synthetase, and changes in muscle mass and quality may lead to elevated circulating ammonia levels, increasing HE risk [35]. Beyond muscle loss, myosteatosis is also associated with chronic liver disease and higher risk of HE in cirrhotic patients [37]. Other studies found an increase in the skeletal muscle index (SMI) > 10% after TIPS compared with baseline (pre-TIPS) to be associated with reduced ammonia levels and a lower prevalence of HE and overt HE episodes compared with patients with an improvement in the SMI < 10% [38]. These results suggest that the nutritional status should be assessed prior to TIPS placement to reduce the incidence of HE.

Our study is primarily limited by its retrospective design. Both baseline and follow-up imaging were not performed at standardized time points but based on clinical indications (e.g., screening for liver tumors, abdominal pain, or complications following TIPS implantation). Therefore, post-TIPS CT scans were not available for many patients, as they are not part of routine follow-up. This may partly explain why no clear trends in body composition after TIPS were observed in our study. Sarcopenia was defined using the lumbar skeletal muscle index, which requires not only CT imaging but also the patient’s height, which was not consistently available in the clinical system. Additionally, patients’ dietary habits and other lifestyle factors were not documented and could therefore not be considered in this retrospective analysis. There was also insufficient documentation of complementary therapies, such as physiotherapy or nutritional counseling after TIPS implantation. Furthermore, criteria for the assessment of sarcopenia are derived from the data of healthy individuals, though evidence suggests that these thresholds may require adjustment for patients with liver cirrhosis [5]. Automated segmentation, which only required verification, offers the possibility to analyze higher numbers of cases in a standardized approach and with comparatively low time expenditure, and thus may be used in large patient populations. In the future, this tool could also be applied prospectively to monitor patients after TIPS in the clinical setting.

## 5. Conclusions

In conclusion, our research highlights the clinical relevance of body composition imaging parameters and their association with postinterventional complications after TIPS, especially the development of hepatic encephalopathy and overall survival. These results underscore the importance of considering body composition and sarcopenia into the clinical management and prognosis of patients undergoing interventions. Sarcopenia can be dynamically monitored using body composition analysis of follow-up CT scans.

## Figures and Tables

**Table 1:** Body composition imaging parameters of the study population at baseline. BMI—Body Mass Index, LSMI—lumbar skeletal muscle index, PMA—psoas muscle area, SAT—subcutaneous adipose tissue, SMA—skeletal muscle area, TAMA—total abdominal muscle area, VAT—visceral adipose tissue.

	No Sarcopenia (n = 60)	Sarcopenia (n = 135)	Total (n = 195)
BMI (Median (IQR))	29.05 (26.1, 33.1)	24.86 (21.9, 28.6)	26.26 (23.03, 30.0)
TAMA (Median (IQR))	158.5 (121.7, 179.8)	113.1(89.1, 137.3)	124.5 (97.2, 147.7)
VAT (Median (IQR))	127.9 (70.6, 196.4)	62.5 (31.5, 121.4)	77.4 (41.5, 150.9)
SMA (Median (IQR))	142.3 (110.5, 160.2)	100.0 (82.4, 122.1)	111.3 (88.4, 131.5)
ATR (Median (IQR))	0.6 (0.4, 0.9)	0.7 (0.4, 1.1)	0.7 (0.4, 1.0)
LSMI (Median (IQR))	54.0 (45.0, 60.1)	37.4 (31.6, 43.7)	41.8 (33.9, 49.7)
PMA (Median (IQR))	14.7 (11.1, 20.0)	11.2 (7.8, 14.9)	12.2 (8.6, 15.8)
SAT (Median (IQR))	211.4 (147.1, 289.3)	90.1 (54.4, 164.9)	132.8 (63.1, 212.6)

**Table 2:** Synopsis of descriptive analysis based on outcome parameters. HE—hepatic encephalopathy, LSMI—lumbar skeletal muscle index.

Outcome Parameters		Sarcopenia (n = 135)	No Sarcopenia (n = 60)	Sarcopenic Obesity (n = 22)	No Sarcopenic Obesity (n = 171)	LSMI [cm^2^/m^2^] 41.8 (33.9, 49.7)
HE	Yes	36 (26.7%)	10 (16.7%	7 (31.8%)	38 (22.2%)	41.70 (34.48, 48.26)
	No	99 (73.3%)	50 (83.3%)	15 (68.2%)	133 (77.8%)	42.03 (33.85, 49.83)
Complication	Yes	60 (44.4%)	17 (28.3%)	8 (36.4%)	67 (39.2%)	40.61 (31.62, 49.59)
	No	75 (55.6%)	43 (71.7%)	14 (63.6%)	104 (60.8%)	42.56 (36.23, 49.78)
TIPS dysfunction	Yes	28 (20.7%)	16 (26.7%)	9 (40.9%)	34 (19.9%)	42.49 (33.74, 52.50)
	No	107 (79.3%)	44 (73.3%)	13 (59.1%)	137 (80.1%)	41.59 (34.23, 48.26)
Death	Yes	34 (25.2%)	13 (21.7%)	3 (13.6%)	44 (25.7%)	42.64 (36.90, 50.16)
	No	101 (74.8%)	47 (78.3%)	19 (86.4%)	127 (74.3%)	41.51 (33.69, 49.62)

## Data Availability

Data available on request due to data protection.

## Abbreviations

AI	Artificial intelligence
ATR	Adipose tissue ratio
BMI	Body Mass Index
CI	Confidence interval
CT	Computed tomography
DICOM	Digital Imaging and Communications in Medicine
HE	Hepatic encephalopathy
HR	Hazard ratio
LSMI	Lumbar skeletal muscle index
PACS	Picture archiving and communication system
PMA	Psoas muscle area
PPG	Portal Pressure Gradient
SAT	Subcutaneous adipose tissue
SF	Subcutaneous Fat Area
SFT	Subcutaneous Fat Thickness
SMA	Skeletal muscle area
SMI	Skeletal muscle index
TAMA	Total abdominal muscle area
TIPS	Transjugular intrahepatic portosystemic shunt
VAT	Visceral adipose tissue

## References

[1] Rössle M., Richter G.M., Noeldge G., Siegerstetter V., Palmaz J.C., Wenz W., Gerok W. (1989). The intrahepatic portosystemic shunt. Initial clinical experiences with patients with liver cirrhosis. Dtsch. Med. Wochenschr..

[2] Schultheiß M., Bettinger D., Thimme R., Rössle M. (2020). 30 Years of Transjugular Intrahepatic Portosystemic Shunt (TIPS): Casting a retrospective glance and future perspectives. Z. Gastroenterol..

[3] Tsien C., Shah S.N., McCullough A.J., Dasarathy S. (2013). Reversal of sarcopenia predicts survival after a transjugular intrahepatic portosystemic stent. Eur. J. Gastroenterol. Hepatol..

[4] Ronald J., Bozdogan E., Zaki I.H., Kappus M.R., Choi S.S., Martin J.G., Suhocki P.V., Smith T.P., Kim C.Y., Bashir M.R. (2019). Relative Sarcopenia With Excess Adiposity Predicts Survival After Transjugular Intrahepatic Portosystemic Shunt Creation. Am. J. Roentgenol..

[5] Liu J., Ma J., Yang C., Chen M., Shi Q., Zhou C., Huang S., Chen Y., Wang Y., Li T. (2022). Sarcopenia in Patients with Cirrhosis after Transjugular Intrahepatic Portosystemic Shunt Placement. Radiology.

[6] Nardelli S., Bellafante D., Ridola L., Faccioli J., Riggio O., Gioia S. (2023). Prevention of post-tips hepatic encephalopathy: The search of the ideal candidate. Metab. Brain. Dis..

[7] Periyalwar P., Dasarathy S. (2012). Malnutrition in cirrhosis: Contribution and consequences of sarcopenia on metabolic and clinical responses. Clin. Liver Dis..

[8] Norman K., Kirchner H., Lochs H., Pirlich M. (2006). Malnutrition affects quality of life in gastroenterology patients. World J. Gastroenterol..

[9] Fehrenbach U., Wuensch T., Gabriel P., Segger L., Yamaguchi T., Auer T.A., Beetz N.L., Denecke C., Kröll D., Raakow J. (2021). CT Body Composition of Sarcopenia and Sarcopenic Obesity: Predictors of Postoperative Complications and Survival in Patients with Locally Advanced Esophageal Adenocarcinoma. Cancers.

[10] Beetz N.L., Geisel D., Maier C., Auer T.A., Shnayien S., Malinka T., Neumann C.C.M., Pelzer U., Fehrenbach U. (2022). Influence of Baseline CT Body Composition Parameters on Survival in Patients with Pancreatic Adenocarcinoma. J. Clin. Med..

[11] Dahlmann S., Bressem K., Bashian B., Ulas S.T., Rattunde M., Busch F., Makowski M.R., Ziegeler K., Adams L. (2023). Sex Differences in Renal Cell Carcinoma: The Importance of Body Composition. Ann. Surg. Oncol..

[12] Beetz N.L., Geisel D., Shnayien S., Auer T.A., Globke B., Öllinger R., Trippel T.D., Schachtner T., Fehrenbach U. (2022). Effects of Artificial Intelligence-Derived Body Composition on Kidney Graft and Patient Survival in the Eurotransplant Senior Program. Biomedicines.

[13] Beetz N.L., Maier C., Shnayien S., Trippel T.D., Gehle P., Fehrenbach U., Geisel D. (2021). Artificial intelligence-based analysis of body composition in Marfan: Skeletal muscle density and psoas muscle index predict aortic enlargement. J. Cachexia Sarcopenia Muscle.

[14] Miljkovic I., Vella C.A., Allison M. (2021). Computed Tomography-Derived Myosteatosis and Metabolic Disorders. Diabetes Metab. J..

[15] Cruz-Jentoft A.J., Bahat G., Bauer J., Boirie Y., Bruyère O., Cederholm T., Cooper C., Landi F., Rolland Y., Sayer A.A. (2019). Sarcopenia: Revised European consensus on definition and diagnosis. Age Ageing.

[16] (2019). EASL Clinical Practice Guidelines on nutrition in chronic liver disease. J. Hepatol..

[17] Büttner L., Aigner A., Pick L., Brittinger J., Steib C.J., Böning G., Streitparth F. (2022). 25 years of experience with transjugular intrahepatic portosystemic shunt (TIPS): Changes in patient selection and procedural aspects. Insights Imaging.

[18] Su H., Ruan J., Chen T., Lin E., Shi L. (2019). CT-assessed sarcopenia is a predictive factor for both long-term and short-term outcomes in gastrointestinal oncology patients: A systematic review and meta-analysis. Cancer Imaging.

[19] Prado C.M., Lieffers J.R., McCargar L.J., Reiman T., Sawyer M.B., Martin L., Baracos V.E. (2008). Prevalence and clinical implications of sarcopenic obesity in patients with solid tumours of the respiratory and gastrointestinal tracts: A population-based study. Lancet Oncol..

[20] Therneau T.M., Grambsch P.M. (2000). The Cox Model. Modeling Survival Data: Extending the Cox Model.

[21] Therneau T. (2023). A Package for Survival Analysis in R.

[22] Wickham H., Averick M., Bryan J., Chang W., McGowan L.D.A., François R., Grolemund G., Hayes A., Henry L., Hester J. (2019). Welcome to the Tidyverse. J. Open Source Softw..

[23] R-Core-Team (2023). R: A Language and Environment for Statistical Computing.

[24] Plauth M., Schütz T., Buckendahl D.P., Kreymann G., Pirlich M., Grüngreiff S., Romaniuk P., Ertl S., Weiss M.L., Lochs H. (2004). Weight gain after transjugular intrahepatic portosystemic shunt is associated with improvement in body composition in malnourished patients with cirrhosis and hypermetabolism. J. Hepatol..

[25] Pang N., Zhao C., Li J., Li L., Yang X., Yang M., Wu Z., Feng D. (2021). Body mass index changes after transjugular intrahepatic portosystemic shunt in individuals with cirrhosis. Nutrition.

[26] Cheung K., Lee S.S., Raman M. (2012). Prevalence and mechanisms of malnutrition in patients with advanced liver disease, and nutrition management strategies. Clin. Gastroenterol. Hepatol..

[27] Allard J.P., Chau J., Sandokji K., Blendis L.M., Wong F. (2001). Effects of ascites resolution after successful TIPS on nutrition in cirrhotic patients with refractory ascites. Am. J. Gastroenterol..

[28] Montano-Loza A.J., Meza-Junco J., Prado C.M., Lieffers J.R., Baracos V.E., Bain V.G., Sawyer M.B. (2012). Muscle wasting is associated with mortality in patients with cirrhosis. Clin. Gastroenterol. Hepatol..

[29] Ebadi M., Bhanji R.A., Tandon P., Mazurak V., Baracos V.E., Montano-Loza A.J. (2020). Review article: Prognostic significance of body composition abnormalities in patients with cirrhosis. Aliment. Pharmacol. Ther..

[30] Praktiknjo M., Book M., Luetkens J., Pohlmann A., Meyer C., Thomas D., Jansen C., Feist A., Chang J., Grimm J. (2018). Fat-free muscle mass in magnetic resonance imaging predicts acute-on-chronic liver failure and survival in decompensated cirrhosis. Hepatology.

[31] Kang S.H., Jeong W.K., Baik S.K., Cha S.H., Kim M.Y. (2018). Impact of sarcopenia on prognostic value of cirrhosis: Going beyond the hepatic venous pressure gradient and MELD score. J. Cachexia Sarcopenia Muscle.

[32] Benmassaoud A., Roccarina D., Arico F., Leandro G., Yu B., Cheng F., Yu D., Patch D., Tsochatzis E. (2020). Sarcopenia Does Not Worsen Survival in Patients With Cirrhosis Undergoing Transjugular Intrahepatic Portosystemic Shunt for Refractory Ascites. Am. J. Gastroenterol..

[33] Praktiknjo M., Clees C., Pigliacelli A., Fischer S., Jansen C., Lehmann J., Pohlmann A., Lattanzi B., Krabbe V.K., Strassburg C.P. (2019). Sarcopenia Is Associated With Development of Acute-on-Chronic Liver Failure in Decompensated Liver Cirrhosis Receiving Transjugular Intrahepatic Portosystemic Shunt. Clin. Transl. Gastroenterol..

[34] Baumann B.C., Wei J., Plastaras J.P., Lukens J.N., Damjanov N., Hoteit M., Hsu C., Levine M., Mondschein J., Nadolski G. (2018). Stereotactic Body Radiation Therapy (SBRT) for Hepatocellular Carcinoma: High Rates of Local Control With Low Toxicity. Am. J. Clin. Oncol..

[35] Merli M., Giusto M., Lucidi C., Giannelli V., Pentassuglio I., Di Gregorio V., Lattanzi B., Riggio O. (2013). Muscle depletion increases the risk of overt and minimal hepatic encephalopathy: Results of a prospective study. Metab. Brain Dis..

[36] Nardelli S., Lattanzi B., Torrisi S., Greco F., Farcomeni A., Gioia S., Merli M., Riggio O. (2017). Sarcopenia Is Risk Factor for Development of Hepatic Encephalopathy After Transjugular Intrahepatic Portosystemic Shunt Placement. Clin. Gastroenterol. Hepatol..

[37] Bhanji R.A., Moctezuma-Velazquez C., Duarte-Rojo A., Ebadi M., Ghosh S., Rose C., Montano-Loza A.J. (2018). Myosteatosis and sarcopenia are associated with hepatic encephalopathy in patients with cirrhosis. Hepatol. Int..

[38] Gioia S., Merli M., Nardelli S., Lattanzi B., Pitocchi F., Ridola L., Riggio O. (2019). The modification of quantity and quality of muscle mass improves the cognitive impairment after TIPS. Liver Int..

